# Maintaining care delivery for non-communicable diseases in the face of the COVID-19 pandemic in western Kenya

**DOI:** 10.11604/pamj.2021.39.143.29708

**Published:** 2021-06-22

**Authors:** Jemima Kamano, Violet Naanyu, Richard Ayah, Obed Limo, Gladwell Gathecha, Eugene Saenyi, Pendo Jefwa, Kenneth Too, Imran Manji, Pooja Gala, Rajesh Vedanthan

**Affiliations:** 1 Department of Medicine, School of Medicine, College of Health Sciences, Moi University, Eldoret, Kenya,; 2 Academic Model Providing Access to Health Care, Eldoret, Kenya,; 3 Department of Sociology Psychology and Anthropology, School of Arts and Social Sciences, Moi University, Eldoret, Kenya,; 4 School of Public Health, College of Health Sciences, University of Nairobi, Nairobi, Kenya,; 5 Division of Non-Communicable Diseases, Ministry of Health, Nairobi, Kenya,; 6 Directorate of Pharmacy and Nutrition, Moi Teaching and Referral Hospital, Eldoret, Kenya,; 7 Department of Medicine, NYU Grossman School of Medicine, New York, USA,; 8 Department of Population Health, NYU Grossman School of Medicine, New York, USA

**Keywords:** Community health, primary health care, COVID-19, diabetes, hypertension, global health

## Abstract

The coronavirus disease 2019 (COVID-19) pandemic has disrupted health systems worldwide, gravely threatening continuity of care for non-communicable diseases (NCDs), particularly in low-resource settings. We describe our efforts to maintain the continuity of care for patients with NCDs in rural western Kenya during the COVID-19 pandemic, using a five-component approach: 1) Protect: protect staff and patients; 2) Preserve: ensure medication availability and clinical services; 3) Promote: conduct health education and screenings for NCDs and COVID-19; 4) Process: collect process indicators and implement iterative quality improvement; and 5) Plan: plan for the future and ensure financial risk protection in the face of a potentially overwhelming health and economic catastrophe. As the pandemic continues to evolve, we must continue to pursue new avenues for improvement and expansion. We anticipate continuing to learn from the evolving local context and our global partners as we proceed with our efforts.

## Commentary

Given the enormity of the health system response required for COVID-19 preparedness and management, as well as the physical distancing measures that impact in-person clinical visits, continuity of care for non-communicable diseases (NCDs) is gravely threatened worldwide, particularly in low-resource settings [1]. In Kenya, COVID-19 found an already highly vulnerable healthcare system that is struggling to address both infectious diseases and NCDs. In this paper, we describe our effort to maintain NCD clinical care delivery during the COVID-19 outbreak within the context of limited resources in western Kenya.

**The primary integrated care for four chronic diseases (PIC4C) project:** the Academic Model Providing Access to Healthcare (AMPATH) program is an academic global health partnership between Moi University College of Health Sciences, Moi Teaching and Referral Hospital, and a consortium of North American universities led by Indiana University [2]. In recognition of the growing NCD burden in Kenya, AMPATH has established a chronic disease management program in collaboration with the Kenya Ministry of Health (MOH), operating in over 150 health facilities spanning all levels of the public sector health care system in western Kenya [3]. Over the past decade, the program has provided multicomponent care to over 40,000 individuals with hypertension and/or diabetes, grounded in the following principles: partnership with communities and governments [4], geographic decentralization of care, task redistribution [5], continuous supply of medications [6], group medical visits [7], and incorporation of social determinants of health into care delivery [8].

As our work has evolved, we have expanded clinical services to include cancer, and have developed an integrated care platform for NCDs. The Primary Integrated Care for Four Chronic Diseases (PIC4C) project, an integrated primary care program for the prevention and control of diabetes, hypertension, breast and cervical cancer, is being implemented in partnership with the Kenyan MOH Division of NCDs in two counties in western Kenya (Busia and Trans Nzoia) [9]. At the current time, over 13,000 patients are receiving care through the PIC4C program in the two counties.

**Primary integrated care for four chronic diseases in the era of COVID-19:** in mid-March 2020, the government of Kenya imposed bans on group meetings and public gatherings; by the end of March, individual movement was restricted and a curfew was instated. In addition, in-person home visits by community health workers (CHWs) were suspended, and non-emergency outpatient clinical services at most health facilities were halted. Transportation costs increased by approximately 50% (range 25-100%) during this time, increasing the challenges for patients to travel to health facilities for clinic appointments. All of these factors threatened the ability of patients to receive timely, comprehensive NCD care. In response, the PIC4C project initiated a series of agile, real-time, and iteratively evolving interventions, in order to maintain continuity of NCD care for our patients in the setting of the COVID-19 pandemic: Protect, Preserve, Promote, Process, and Plan.

**Figure 1:**
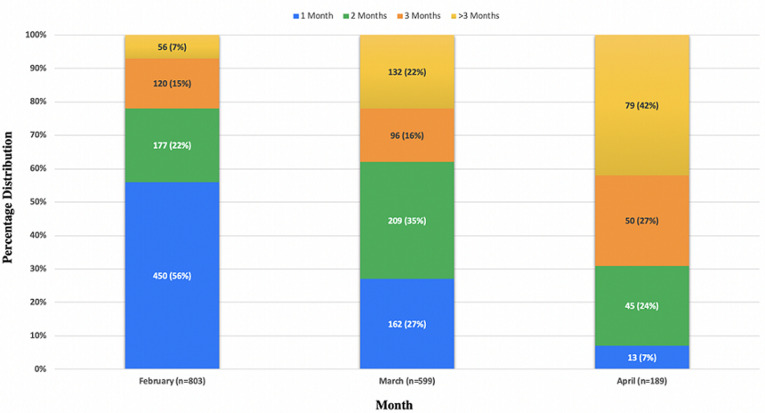
distribution of length to follow-up appointments for patients with diabetes seen from Feb-April 2020

**Protect:** we initiated measures to protect clinicians, staff, and patients. We set up hand washing equipment and supplies in all of the health facilities supported by PIC4C. We also provided our clinicians and CHWs with surgical masks, hand washing equipment/hand sanitizer, and lab coats. We shifted to open-air education sessions and screening activities, and attendees were issued masks. To decongest clinics, we limited in-person clinical encounters to patients needing urgent evaluation. Stable patients were given return appointments at least three months later instead of one month. Figure 1 summarizes return-to-clinic data from the AMPATH medical records system and demonstrates that there was an increasing proportion of patients receiving longer length of follow-up visits over time. Patients were offered medication supplies for the entire duration until their next appointment, including free insulin for insulin-dependent patients.

**Preserve:** preserving the services available to our NCD patients was of utmost priority. We focused on strengthening the medication supply chain to ensure sufficient medication stocks throughout our catchment area [10]. We established decentralized warehouses in the peripheral health facilities instead of relying solely on the central pharmacy at the referral hospital. We instituted a system of “medicine tackle boxes” to stock the smallest health facilities in very rural areas.

**Figure 2:**
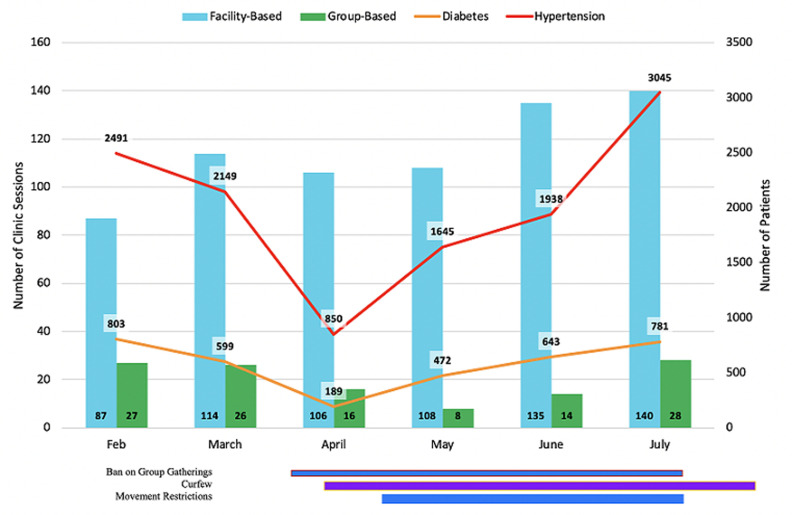
number of clinic sessions (vertical bars) and patients seen (line graph) at PIC4C facilities from February to July 2020; solid horizontal bars indicate periods of COVID-19 public measures put into place by the government of Kenya; a ban on group gatherings was instated from March 15-July 7; curfew was enforced from March 27 and is ongoing; restrictions on movement started on April 6 and was lifted in phases starting July 7

We also ensured that clinicians were available to conduct both in-person and phone clinical consultations with our patients. Clinic visit and patient encounter data from the AMPATH medical records system demonstrate that there was a temporary decline in clinic sessions and patients seen (Figure 2), which can be attributed to multiple factors: COVID-related policies limiting movement and gatherings, increased cost of transportation, and the increased time interval for follow-up visits, as described above. Additional barriers likely included fear of contracting COVID-19, school closures requiring childcare arrangements, and economic challenges. Despite these barriers, the clinics were maintained during this time period for patients requiring urgent care visits.

To preserve patient health, we also responded to other concurrent community stressors. For instance, Busia county experienced heavy flooding that caused severe disruption to food supply and livelihoods of many of our patients. We responded by providing monetary support, food, clothing, and transportation. We also provided a one-month free supply of hypertension and diabetes medications to patients affected by the floods.

**Figure 3:**
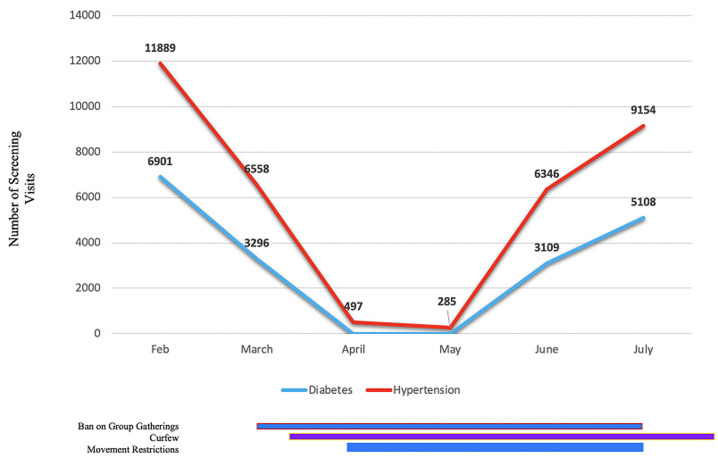
number of screening visits for hypertension and diabetes from February to July 2020; solid horizontal bars indicate periods of COVID-19 public measures put into place by the Government of Kenya; a ban on group gatherings was instated from March 15-July 7; curfew was enforced from March 27 and is ongoing; restrictions on movement started on April 6 and was lifted in phases starting July 7

**Promote:** we took steps to promote health screening, education, and appropriate referrals. We initially conducted an intensive telephone outreach effort to contact our patients with diabetes or hypertension. Starting in late March, among the 5,457 patients who had provided our program with phone numbers, 3,992 (73%) were successfully contacted, of whom 3,855 (97%) received health education on COVID-19. We also disseminated health education via nine radio shows reaching a catchment population of 1,892,647 people from February to July 2020. As the restrictions on in-person gatherings were gradually relaxed, we resumed community screenings for hypertension, diabetes, cervical cancer, and breast cancer in June-July 2020. Data from the PIC4C project database demonstrate that, within two months, we were able to resume the same level of screenings as were achieved before the COVID-19 pandemic (Figure 3).

**Process:** we strove to evaluate the quality and process indicators of the services we provided during this period of time. Patient feedback indicated that the COVID-19 health education was well received, and that contact from the PIC4C team was appreciated. There was positive feedback regarding the availability of drugs closer to home at local clinics with affordable prices. We learned about the importance of engaging facility and local leadership at all phases of implementing our activities, which was crucial in ensuring that all COVID-19 prevention regulations were being adhered to during community activities.

However, there were also some notable concerns and complaints. First, patients reported being unable to use their national health insurance fund (NHIF) coverage for medication procurement at the lower tiers of care closer to their homes, despite having paid their premiums, which posed a medication access challenge for NCD patients. Second, some patients reported that clinical staff were unfriendly, which negatively impacted continuity of care. Finally, some of our patients were fearful being exposed to COVID-19 and did not venture outside of their homes to seek care even when experiencing cardiovascular complications from uncontrolled disease, such as strokes. We responded to these issues by iteratively modifying and adapting our approach and taking the following steps: 1) We crafted an arrangement with the county health management teams and the pharmacy program to refill medications at higher level facilities and subsequently deliver them to patients through local dispensaries. 2) We increased support supervision of health workers by sub-county management teams with emphasis on customer care and promotion of retention to care. 3) We added episodes to our radio talk show focusing on cardiovascular complications of diabetes and hypertension, emphasizing prevention and early recognition of signs and symptoms of complications. 4) We implemented virtual continuing medical education to clinicians on diabetes and hypertension, as well as emerging mental health issues, in the context of the COVID-19 pandemic. Our process evaluation served as an important tool in improving our services utilizing feedback from key stakeholders including patients, local leaders, and our clinical teams.

**Plan:** in order to plan for the future, we established infrastructure, protocols, and practices that could be utilized in the event of either prolonged or recurrent COVID-19-related disruptions to clinical care. For instance, we began to pilot tele-medicine services using CHWs and peer support as “clinician-extenders”, while considering the reality that a majority of our rural, poor patients do not have cell phones. We also invigorated efforts to ensure financial risk protection in the face of potentially overwhelming health and economic catastrophes. We increased our engagement with microfinance activities, and actively promoted the uptake of NHIF. Finally, we collaborated with county governments to implement COVID-19 preparedness and management, while ensuring the safety of our patients and staff.

## Conclusion

We have summarized the implementation of our efforts to maintain care delivery for NCDs in the face of the COVID-19 pandemic using our five-pronged plan: protect, preserve, promote, process, and plan. As the pandemic continues to evolve, we will also evolve our response. We therefore recognize that we must remain vigilant, evaluate and identify shortcomings, and pursue new avenues for improvement and expansion. Our experiences have been positive, but have also highlighted issues requiring future action. We anticipate continuing to learn from the evolving local context and our global partners as we proceed with our efforts to ensure high quality NCD care in the context of the COVID-19 pandemic.

**Funding information:** funding for the PIC4C project is supported by Access Accelerated through the World Bank grant number World Bank TFA5636 (Case study integrated delivery of selected non-communicable diseases in Kenya: PIC4C). The funding sources had no role in the analysis or presentation of the data and results. The authors affirm that we have not entered into an agreement with the funder that may have limited our ability to complete the research and we have had full control of all primary data.
